# Detection of spacer precursors formed in vivo during primed CRISPR adaptation

**DOI:** 10.1038/s41467-019-12417-w

**Published:** 2019-10-10

**Authors:** Anna A. Shiriaeva, Ekaterina Savitskaya, Kirill A. Datsenko, Irina O. Vvedenskaya, Iana Fedorova, Natalia Morozova, Anastasia Metlitskaya, Anton Sabantsev, Bryce E. Nickels, Konstantin Severinov, Ekaterina Semenova

**Affiliations:** 1 Center of Life Sciences, Skolkovo Institute of Science and Technology, 1 Nobel St., Moscow, 121205 Russia; 2 Peter the Great St. Petersburg Polytechnic University, 29 Polytechnicheskaya St., St. Petersburg, 195251 Russia; 3 Department of Molecular Biology and Biochemistry, Waksman Institute, Rutgers University, 190 Frelinghuysen Rd., Piscataway, NJ 08854 USA; 4 Institute of Molecular Genetics, Russian Academy of Sciences, 2 Akademika Kurchatova Sq., Moscow, 123182 Russia; 5 Department of Genetics, Waksman Institute, Rutgers University, 190 Frelinghuysen Rd., Piscataway, NJ 08854 USA

**Keywords:** Sequencing, CRISPR-Cas systems, DNA metabolism

## Abstract

Type I CRISPR-Cas loci provide prokaryotes with a nucleic-acid-based adaptive immunity against foreign DNA. Immunity involves adaptation, the integration of ~30-bp DNA fragments, termed prespacers, into the CRISPR array as spacers, and interference, the targeted degradation of DNA containing a protospacer. Interference-driven DNA degradation can be coupled with primed adaptation, in which spacers are acquired from DNA surrounding the targeted protospacer. Here we develop a method for strand-specific, high-throughput sequencing of DNA fragments, FragSeq, and apply this method to identify DNA fragments accumulated in *Escherichia coli* cells undergoing robust primed adaptation by a type I-E or type I-F CRISPR-Cas system. The detected fragments have sequences matching spacers acquired during primed adaptation and function as spacer precursors when introduced exogenously into cells by transformation. The identified prespacers contain a characteristic asymmetrical structure that we propose is a key determinant of integration into the CRISPR array in an orientation that confers immunity.

## Abstract (web summary)

Primed adaptation in the CRISPR-Cas system helps recognition of previously encountered sequence elements and promotes the formation of new memories. Here the authors characterized spacer precursors of type I-E and type I-F CRISPR-Cas system using in vivo models.

## Introduction

CRISPR interference in the *Escherichia coli* type I-E system is performed by the Cascade complex, composed of a crRNA and several Cas proteins^1–3^. Initial binding of Cascade to a protospacer flanked by a 3-bp protospacer adjacent motif (PAM)^4^ results in the formation of an R-loop containing an RNA–DNA heteroduplex formed between the crRNA and target strand, and extrusion of single-stranded DNA derived from the nontarget strand^2,5–10^. Cas3, a single-stranded nuclease and 3′–5′ helicase, is recruited to the Cascade–protospacer complex and cleaves the nontarget strand to initiate unwinding and degradation of the targeted DNA^6,10,11^. In vitro, Cas3 can translocate on DNA as a component of a larger complex that includes Cascade and the key proteins of CRISPR adaptation, Cas1 and Cas2 ^12^.

CRISPR adaptation in the *E. coli* I-E system is mediated by a Cas1–Cas2 complex that can facilitate spacer acquisition in the absence of interference, a process termed naive adaptation^13–16^. The Cas1–Cas2 complex incorporates synthetic double-stranded DNA fragments associated with consensus 5′-AAG-3′/3′-TTC-5′ PAM (PAM^AAG^) into the CRISPR array in orientation dictated by the PAM sequence and conferring immunity^17^. However, the state of the natural prespacers captured by Cas1–Cas2 in cells and the mechanism ensuring integration of a prespacer in a specific orientation remains unknown.

In primed CRISPR adaptation, interference-driven DNA degradation initiated at a priming protospacer (PPS) is coupled with acquisition of spacers from DNA in the PPS region^18–20^. One hallmark of primed adaptation is that nearly all PPS-region sequences from which spacers are acquired contain a consensus PAM^AAG^^18–20^. A second hallmark of primed adaptation is that spacer acquisition occurs in a bidirectional, orientation-dependent manner relative to the PAM of the PPS. In particular, the non-transcribed strand of spacers acquired from the PAM-proximal region (upstream) or PAM-distal region (downstream) is derived from the nontarget strand or target strand, respectively^21^. Available in vivo models of primed adaptation that contain a plasmid-borne PPS or phage-borne PPS are limited due to difficulties in detecting bidirectional spacer acquisition or by high rates of cell lysis^18,19,21^. In particular, analysis of spacer acquisition from circular targets, especially small plasmids, is complicated due to overlapping gradients of protospacers located both upstream and downstream of the PPS^18,19,21^. Use of long linear PPS-containing phage genomes imposes difficulties associated with phage biology such as the inability to detect adaptation for some phages or high rates of cell lysis caused by the others^21^.

Here we construct a robust in vivo model for primed adaptation consisting of an *E. coli* type I-E CRISPR–Cas self-targeting locus encoding a crRNA that targets a chromosomal protospacer. We develop a strand-specific, high-throughput sequencing method for analysis of DNA fragments, FragSeq, and use this method to detect short fragments derived from the DNA surrounding the targeted protospacer. The detected fragments have sequences matching spacers acquired during primed adaptation, contain ~3- to 4-nt overhangs derived from excision of genomic DNA within a PAM, are generated in a bidirectional, orientation-dependent manner relative to the targeted protospacer, require the functional integrity of machinery for interference and adaptation to accumulate, and function as spacer precursors when introduced exogenously into cells by transformation. DNA fragments with a similar structure accumulate in cells undergoing primed adaptation in a type I-F CRISPR–Cas self-targeting system. We propose that the asymmetrical structure of the spacer precursors detected in this work is a key determinant of spacer integration into the CRISPR array in orientation conferring immunity.

## Results

### Type I-E self-targeting leads to robust primed adaptation

**Fig. 1:**
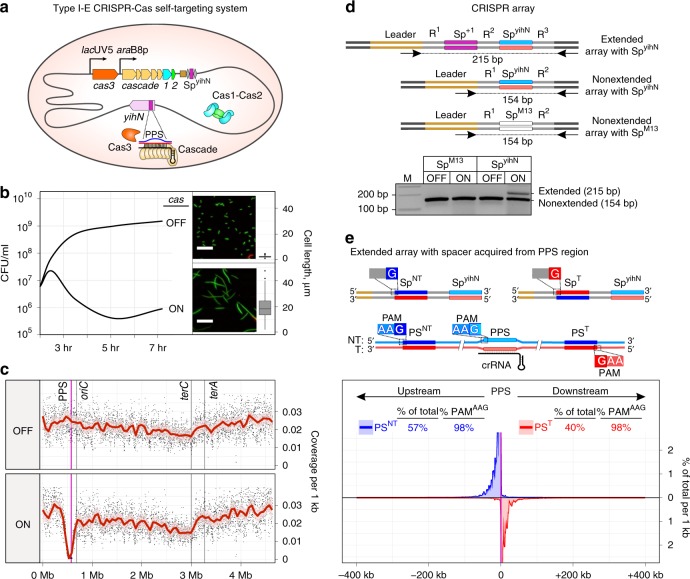
Interference-driven DNA degradation coupled with spacer acquisition in a type I-E self-targeting system. **a** Type I-E self-targeting system. Shaded oval, *E. coli* cell; gray line, chromosome; orange, tan, blue, and green pentagons, *cas* genes; brown rectangle, CRISPR-array leader sequence; gray diamonds, repeats; purple rectangle, spacer targeting *yihN* (Sp^yihN^); *lac*UV5 and *ara*B8p, promoters; mauve pentagon, *yihN*; PPS, priming protospacer within *yihN*; blue line, nontarget strand; red line, target strand; black line, crRNA. **b** Effect of self-targeting on cell growth. Growth curve for cultures in which *cas* gene expression is induced (ON) or not induced (OFF). Green, viable cells; red, non-viable cells; scale bar, 20 μm. Boxplot: the central line, median; hinges, the first and third quartiles; whiskers, 1.5 × IQR; *n* = 125. **c** Effect of self-targeting on genomic DNA content. *oriC*, site of replication origin; *terA* and *terC*, sites of replication termination; dot, coverage per 1 kb; red line, Loess smoothing; pink shading, 99% confidence interval. **d** Effect of self-targeting on spacer acquisition: PCR. Schematics depict an extended array containing Sp^yihN^ and acquired spacer Sp^+1^, a nonextended array containing Sp^yihN^, or an array containing a spacer targeting M13 phage (Sp^M13^). Blue line, non-transcribed strand of Sp^yihN^; red line, transcribed strand of Sp^yihN^ (directs synthesis of crRNA); R, repeats. Arrows below arrays represent the positions of primers used in PCR; sizes of PCR amplicons are indicated. Results show PCR analysis of cells containing an array with Sp^yihN^ or Sp^M13^. M, double-stranded DNA marker. **e** Effect of self-targeting on spacer acquisition: high-throughput sequencing analysis. Top, extended arrays with spacers acquired from PPS-region protospacers. Bottom, results. Sp^NT^, spacer with non-transcribed strand derived from nontarget strand (NT, blue) and transcribed strand derived from target strand (T, red); Sp^T^, spacer with non-transcribed strand derived from target strand (T, red) and transcribed strand derived from nontarget strand (NT, blue). PS^NT^, protospacer for Sp^NT^; PS^T^, protospacer for Sp^T^. Plot shows percentage of spacers per 1 kb derived from PS^NT^ (blue) or PS^T^ (red). Mean of three biological replicates is shown. Source data are provided as a Source Data file

To overcome limitations of primed adaptation systems with plasmid-borne PPS or phage-borne PPS, we constructed a derivative of *E. coli* K12 with a type I-E CRISPR–Cas locus containing a spacer, Sp^yihN^, encoding a crRNA targeting a chromosomal protospacer in the non-essential gene *yihN* (Fig. 1a; Supplementary Table 1). Induction of *cas* gene expression in self-targeting cells leads to inhibition of cell growth accompanied by an increase in cell length (Fig. 1b). Furthermore, analysis of chromosomal DNA by high-throughput sequencing shows that induction of *cas* gene expression causes a dramatic loss of ~300 kb of chromosomal DNA in the PPS region (Fig. 1c, Supplementary Fig. 1a, b, Supplementary Table 2). Loss of PPS-region DNA is also observed in cells containing a catalytically inactive Cas1 variant (Cas1^H208A^)^22^ but is not observed in cells containing a nuclease-deficient Cas3 variant (Cas3^H74A^)^10^ or cells in which Sp^yihN^ is replaced by a spacer targeting M13 phage (Sp^M13^)^9^ (Supplementary Fig. 1a, Supplementary Table 3). Similar results are obtained using methods for analysis of double-stranded or single-stranded DNA (Supplementary Fig. 1b, Supplementary Table 2), indicating that interference-driven degradation of both the target and nontarget strands occurs in the self-targeting strain. The results establish that induction of *cas* gene expression results in interference-driven degradation of PPS-region DNA in the type I-E CRISPR–Cas self-targeting system.

To determine whether interference-driven degradation of PPS-region DNA is coupled with spacer acquisition from PPS-region sequences, we analyzed CRISPR arrays by PCR (Fig. 1d). Results indicate that ~20% of arrays acquire a spacer in cells in which *cas* gene expression is induced, while no spacer acquisition is detected in cells in which *cas* gene expression is not induced (Fig. 1d). Furthermore, no spacer acquisition is detected in cells in which Sp^yihN^ is replaced by Sp^M13^ (Fig. 1d), indicating that spacer acquisition requires interference-driven degradation of PPS-region DNA. High-throughput sequencing analysis of amplicons derived from arrays that have acquired a spacer indicate that the self-targeting system exhibits the defining hallmarks of primed adaptation. In particular, >95% of spacers are acquired from a PAM^AAG^-containing protospacer in the PPS region and, furthermore, spacer acquisition occurs in a bidirectional, orientation-dependent manner characteristic of the *E. coli* I-E system^21^ (Fig. 1e, Supplementary Tables 4, 5). We conclude that the type I-E CRISPR–Cas self-targeting strain provides a robust in vivo model system for primed adaptation.

### FragSeq detects PPS-region-derived fragments

**Fig. 2:**
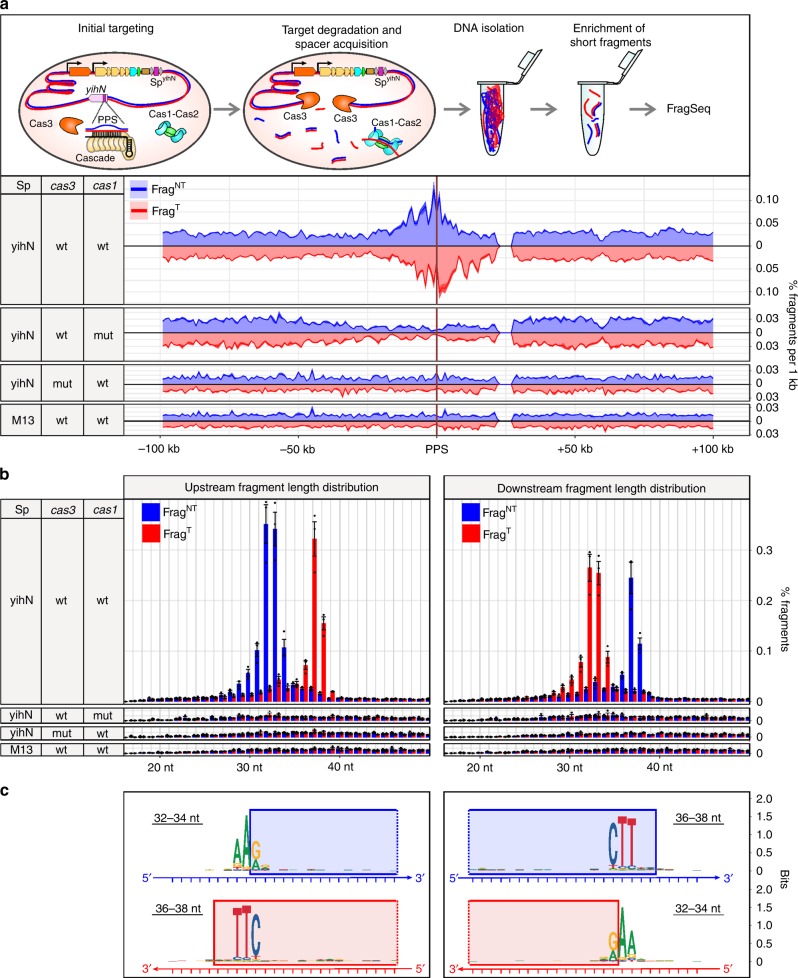
FragSeq detection of DNA fragments in cells with the type I-E self-targeting system. **a** Effect of self-targeting on PPS-region DNA fragment distributions. Top, events occurring in cells upon induction of *cas* gene expression. Bottom, FragSeq results. Coverage plots show mean of three biological replicates. Blue, nontarget-strand-derived fragments (Frag^NT^); red, target-strand-derived fragments (Frag^T^). **b** Length distributions of PPS-region-derived fragments (mean ± SEM of three biological replicates). **c** Sequence alignments of genomic DNA from which PPS-region fragments are derived. Blue rectangles, sequences present in Frag^NT^; red rectangles, sequences present in Frag^T^. Source data are provided as a Source Data file

It has been proposed that interference-driven DNA degradation produces fragments that serve as spacer precursors in primed adaptation^19,23^. To test this model, we developed a method for strand-specific, high-throughput sequencing of DNA fragments, FragSeq. To perform FragSeq, we isolated genomic DNA fragments <700 bp in length, denatured the fragments, ligated single-stranded adapters to the 5′ and 3′ ends of the fragments, amplified the ligation products by PCR, and analyzed the sequences of the fragments by high-throughput sequencing. Because the library construction steps in FragSeq do not involve tailing—i.e., the addition of non-templated nucleotides onto fragment ends—the 5′- and 3′-end sequences of the fragments can be identified with single-nucleotide resolution. We applied FragSeq to identify products of degradation in self-targeting cells undergoing primed adaptation (Fig. 2a, Supplementary Figs. 2–4, Supplementary Tables 6–12 and Methods). Results show accumulation of fragments derived from PPS-region DNA in wild-type cells but not in cells containing inactive variants of Cas1 or Cas3, or cells in which Sp^yihN^ is replaced by Sp^M13^ (Fig. 2a, Supplementary Fig. 3a, Supplementary Table 7). Thus, accumulation of PPS-region-derived fragments in cells undergoing primed adaptation requires the functional integrity of both interference and adaptation.

Analysis of length distributions of the PPS-region-derived fragments indicates that they are produced in a bidirectional, orientation-dependent manner reminiscent of spacer acquisition (Fig. 2b). The most abundant nontarget-strand fragments (Frag^NT^) and target-strand fragments (Frag^T^) emanating from the PAM-proximal region of the PPS (upstream) are 32- to 34-nt and 36- to 38-nt, respectively, and the most abundant Frag^NT^ and Frag^T^ emanating from the PAM-distal region of the PPS (downstream) are 36- to 38-nt and 32- to 34-nt, respectively (Fig. 2b). In addition, the relative abundance of complementary 32- to 34-nt and 36- to 38-nt fragments shows a positive correlation (Pearson correlation coefficient 0.48, Supplementary Table 11), suggesting that the fragments identified by FragSeq represent individual strands of double-stranded DNA products having lengths similar to that of spacers (~30 bp). Alignments of the chromosomal sequences associated with the 5′ or 3′ ends of complementary fragments reveals the presence of a consensus 5′-AAG-3′/3′-TTC-5′ PAM derived from sequences associated with the 5′ ends of 32- to 34-nt fragments and the 3′ ends of 36- to 38-nt fragments (Fig. 2c, Supplementary Tables 9, 10). Thus, the results of FragSeq suggest that cells undergoing primed adaptation accumulate 33- or 34-bp double-stranded DNA fragments containing a 3′ end, 4- or 3-nt overhang derived from excision of a PAM-containing sequence (Fig. 2c). Furthermore, the relative abundance of these fragments and spacers acquired during primed adaptation that have an identical sequence shows a positive correlation (Pearson correlation coefficient 0.5–0.6, Supplementary Table 12). Accordingly, the results strongly suggest the fragments accumulating in cells undergoing primed adaptation are products of an intermediate step between protospacer selection and spacer integration.

### PPS-region-derived fragments function as prespacers

**Fig. 3:**
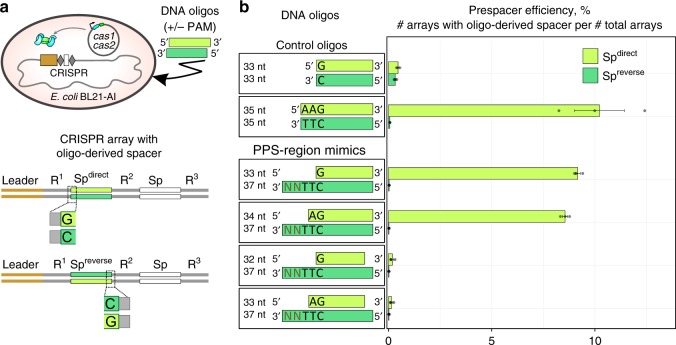
Synthetic mimics of DNA fragments detected in cells undergoing primed adaptation function as prespacers. **a** Prespacer efficiency assay. Top, introduction of synthetic DNA into cells containing a CRISPR array and plasmid that directs expression of *cas1* and *cas2*. Bottom, integration of synthetic DNA into the CRISPR array occurs in either a direct (Sp^direct^) or reverse (Sp^reverse^) orientation. **b** Results. Left, oligonucleotides analyzed. Right, percentage of arrays containing oligo-derived spacers having a direct (light green) or reverse (dark green) orientation (mean ± SEM of three biological replicates). Source data are provided as a Source Data file

To directly test whether the PPS-region-derived fragments detected by FragSeq serve as substrates for spacer integration, we performed a prespacer efficiency assay^17^ (Fig. 3a). We tested synthetic mimics corresponding to the most abundant PPS-region-derived fragments (Fig. 3b, Supplementary Tables 13–16). Results show that 33- or 34-bp synthetic mimics containing a 3′-end, 4- or 3-nt overhang on the PAM-derived end, respectively, and a blunt PAM-distal end were integrated into arrays with an efficiency similar to a control fragment containing a consensus PAM^AAG^ (~10% prespacer efficiency; Fig. 3b, Supplementary Tables 14, 15). In addition, the synthetic mimics and PAM^AAG^-containing control fragment were integrated in a direct orientation with the G:C of the PAM positioned adjacent to the first repeat in the array (Fig. 3, Supplementary Table 15). Introduction of a 5′-end, 1-nt overhang on the PAM-distal end reduced prespacer efficiency by ~45-fold (Fig. 3b, Supplementary Table 15). The results establish that PPS-region-derived fragments containing a 3′-end overhang on the PAM-derived end and blunt PAM-distal end function as efficient spacer precursors.

### Prespacers in I-E and I-F systems exhibit similar structures

**Fig. 4:**
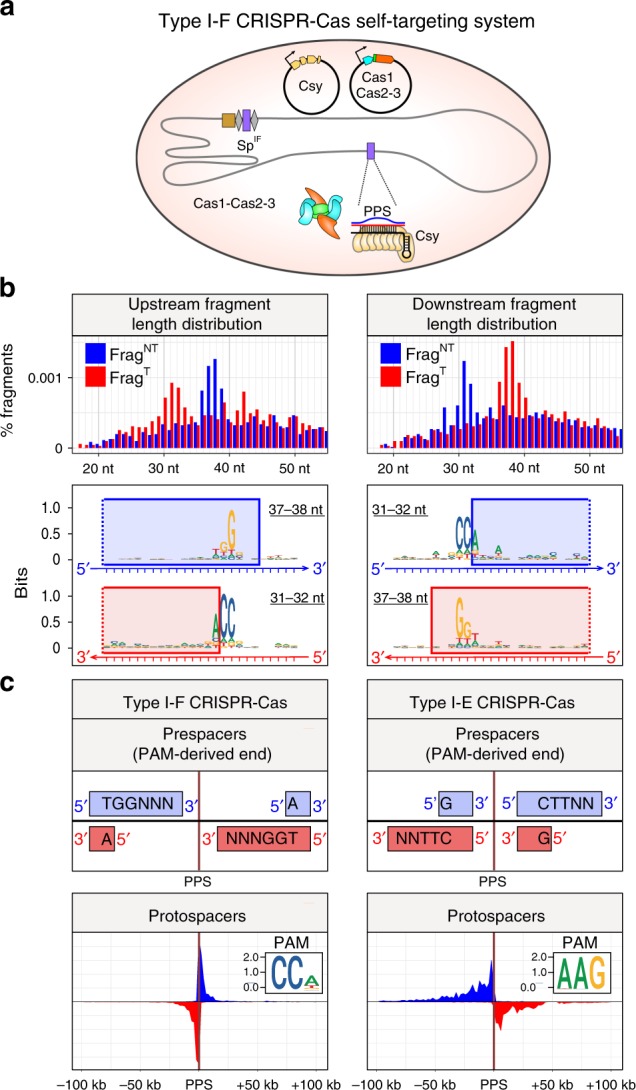
Identification of spacer precursors generated in a type I-F self-targeting system by FragSeq. **a** Components of type I-F CRISPR–Cas self-targeting system. Shaded oval, *E. coli* cell; gray line, chromosomal DNA; black line, plasmid DNA; orange, tan, blue, and green pentagons, *cas* and *csy* genes; brown rectangle, array leader sequence; gray diamonds, array repeat sequences; purple rectangles, spacer and chromosomal PPS targeted by spacer-derived crRNA; Csy, type I-F effector complex; Cas1–Cas2-3, complex of Cas1 and Cas2-3 proteins. **b** FragSeq results: length distributions of fragments (top) and sequence features of PPS-region sequences from which fragments are derived (bottom). Logos for 31–32-nt fragments were generated by aligning sequences 10-nt upstream to 15-nt downstream of the fragment 5′ end. Logos for 37–38-nt fragments were generated by aligning sequences 20-nt upstream to 5-nt downstream of the fragment 3′ end. Blue rectangles, sequences present in Frag^NT^; red rectangles, sequences present in Frag^T^. **c** Comparison of PPS-region-derived fragments and PPS-region protospacers in type I-F and type I-E self-targeting systems. Inset, logo derived from alignment of PPS-region PAMs

In a prior work, we developed an *E. coli* strain that provides a model system for studies of self-targeting by the type I-F CRISPR–Cas system from *Pseudomonas aeruginosa*^24^ (Fig. 4a). Compared with the orientation bias in spacer acquisition observed in type I-E systems, orientation bias in type I-F systems is reversed. In particular, the non-transcribed strand of spacers acquired from the PAM-proximal region of the PPS (upstream) or PAM-distal region of the PPS (downstream) are derived from the target strand or nontarget strand, respectively in type I-F. To determine whether spacer precursors could be detected in the type I-F system, we performed FragSeq analysis in cells undergoing primed adaptation (Fig. 4b, Supplementary Tables 17–21). Similar to the type I-E system, we detect accumulation of spacer-sized double-stranded DNA fragments containing a 3′-end, 5-nt overhang on the PAM-derived end (Fig. 4b). Thus, in spite of exhibiting opposite orientation bias in spacer acquisition, primed adaptation in type I-E and type I-F systems involves generation of spacer precursors with a similar structure (Fig. 4c).

## Discussion

**Fig. 5:**
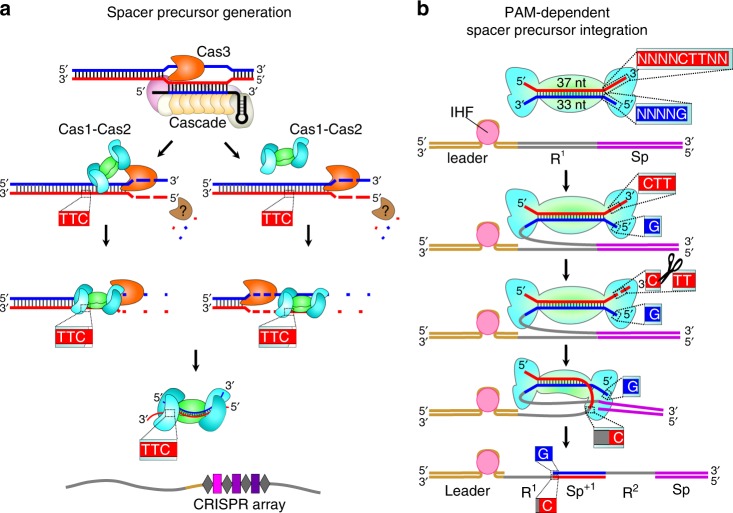
Model of primed adaptation in type I-E CRISPR–Cas systems. **a** Generation of spacer precursors involves coordination between interference and adaptation. Pathway on left depicts direct coordination between interference and adaptation in which Cas1–Cas2 associates with Cas3 as it moves along DNA^12,34^. Pathway on right depicts indirect coordination between interference and adaptation in which Cas1–Cas2 captures products of Cas3-mediated DNA degradation^19,23^. Both pathways generate spacer precursors containing a 3′-end overhang on the PAM-derived end and blunt PAM-distal end. Model depicts rapid degradation of DNA not selected as a spacer precursor by Cas3 and an unknown nuclease (brown). Binding of prespacers to Cas1–Cas2 prevents prespacer degradation. **b** Sequential integration of spacer precursors. First, binding of IHF to the leader stimulates integration of the blunt PAM-distal end between the leader and first repeat sequence^35^. Second, the 3′-overhang present on the PAM-derived end is cleaved by Cas1^36^ or DnaQ exonucleases^37,38^ facilitating integration between the first repeat sequence and first spacer of the array. The order of events depicted results in integration of the spacer precursor in a direct orientation with respect to the PAM (see Fig. 3a)

In summary, we have identified spacer precursors produced as products of an intermediate step (or steps) between protospacer selection and spacer integration for type I-E and type I-F CRISPR–Cas systems. Accumulation of spacer precursors in the type I-E system requires the functional integrity of components of interference and adaptation (Fig. 5) indicating that protospacer selection involves coordination between the interference machinery and adaptation machinery (Fig. 5a). Strikingly, spacer precursors detected during primed adaptation in both type I-E and type I-F systems share an asymmetrical structure characterized by a 3′-end overhang on the PAM-derived end. Thus, we propose that spacer precursors detected in this work are products generated during universal steps of prespacer processing in type I CRISPR–Cas systems relying on Cas1 and Cas2 and lacking auxiliary adaptation proteins. We further propose that the asymmetrical structure of the spacer precursors detected in this work is a key determinant of the sequential integration of prespacers into the CRISPR array (Fig. 5b). In addition, the FragSeq method reported in this work should be applicable, essentially without modification, to identify spacer precursors that form in vivo in any CRISPR–Cas system.

## Methods

### Bacterial strains and plasmids

The *E. coli* strains used in this study are listed in Supplementary Table 1. Red recombinase-mediated gene-replacement technique was used to obtain strains KD403, KD518 and KD753^25^.

Plasmid pCas1 + 2 for the expression of type I-E *cas1* and *cas2* genes as well as plasmids pCas and pCsy for expression type I-F *cas* and *csy* genes were described earlier^13,24^.

### Growth conditions

For analysis of CRISPR-mediated self-targeting by the type I-E system, overnight culture of KD403 strain grown at 37 °C in LB medium was diluted 100-fold into 10 ml of fresh LB and incubated at 37 °C until OD_600_ reached 0.3. The culture was divided into two portions, *cas* genes inducers, IPTG and l-(+)-arabinose were added at 1 mM concentration to one portion, and cultures with and without inducers were incubated at 37 °C for 7 h. At various time points postinduction, the cells were plated with serial dilutions on 1.5% LB agar plates for counting colony forming units (CFUs) or were monitored using fluorescent microscopy.

In assays using strains KD403, KD518, KD753 and KD263 that were followed by sequencing of total genomic DNA, short DNA fragments or newly acquired spacers, similar conditions of culture growth and *cas* genes induction were applied, except that overnight cultures were diluted 100-fold in 100 ml of LB and grown at 30 °C. Five hours postinduction, 10 ml of cells were pelleted by centrifugation at 3000×*g* for 5 min at 4 °C, washed with 10 ml of PBS, pelleted by centrifugation at 3000×*g* for 5 min at 4 °C and resuspended in 1 ml of PBS. The cells were divided into 125-μl aliquots and stored at −70 °C before they were used for DNA isolation.

For analysis of short DNA fragments generated during self-targeting by the type I-F system, cultures of strain KD675 transformed with plasmids pCas and pCsy were grown at 37 °C in LB supplemented with 100 μg/ml ampicillin and 50 μg/ml spectinomycin. Overnight cultures were diluted 200-fold into 10 ml of LB without antibiotics, grown at 37 °C until OD_600_ reached 0.3 and supplemented with 1 mM IPTG and 1mM l-(+)-arabinose. The cells were harvested 24 h postinduction and prepared for DNA isolation as described above for strains KD403, KD518, KD753 and KD263.

### Fluorescence microscopy

Cultures grown with or without induction of *cas* gene expression were analyzed using a LIVE/DEAD viability kit (Thermo Scientific) at 5 h after induction. Viable cells in each culture were detected by addition of 20 μM SYTO9, green fluorescent dye that can penetrate through intact cell membranes. Non-viable cells in each culture were detected by addition of 20 μM propidium iodide dye, which cannot enter viable cells. Sample chambers were made using a microscope slide (Menzel–Gläser) with two strips on the upper and lower edges formed by double-sided sticky tape (Scotch TM). To obtain a flat substrate required for high-quality visualization of bacteria, a 1.5% agarose solution was placed between tape strips and covered with another microscopic slide. After solidification of the agarose, the upper slide was removed and several agarose pads were formed; 1 μl of each cell suspension (with and without induction) was placed on an agarose pad. The microscopic chamber was sealed using a coverslip (24 × 24 mm, Menzel–Gläser).

Fluorescence microscopy was performed using Zeiss AxioImager.Z1 upright microscope. Fluorescence signals in green (living cells) and red (dead cells) fluorescent channels were detected using Zeiss Filter Set 10 and Semrock mCherry-40LP filter set, respectively. Fluorescent images of self-targeting cells were obtained using Cascade II:1024 back-illuminated EMCCD camera (Photometrics). The microscope was controlled using AxioVision Microscopy Software (Zeiss). All image analysis was performed using ImageJ (Fiji) with ObjectJ plugin used for measurements of cell length^26^.

### High-throughput sequencing of total genomic DNA

Total genomic DNA was purified by GeneJET Genomic DNA Purification Kit (Thermo Fisher Scientific). Sequencing libraries were prepared either by NEBNext® Ultra™ II DNA Library Prep Kit for Illumina (NEB) or by Accel-NGS® 1S Plus DNA Library Kit (Swift Biosciences) and sequenced on a NextSeq 500 platform.

Raw reads were analyzed in R with ShortRead and Biostrings packages^27^. Reads with no more than two bases with quality <20 were mapped to the KD403 reference genome using Unipro UGENE platform^28^. Bowtie2 was used as a tool for alignment with end-to-end alignment mode and 1 mismatch allowed^29^. The BAM files were analyzed by Rsamtools package and reads with the MAPQ score equal to 42 were selected and used for downstream coverage analysis^30^. Mean coverage over non-overlapping 1 kb bins was calculated and normalized to the total coverage (the sum of means).

### High-throughput sequencing of newly acquired spacers

Cell lysates were prepared by resuspending cells in water and heating at 95 °C for 5 min. Cell debris was removed from lysates by centrifugation at 16×*g* for 1 min. For the analysis of spacer acquisition in strains KD263 and KD403, lysates were used in PCR reactions containing primers LDR-F2 (ATGCTTTAAGAACAAATGTATACTTTTAG) and Ec_minR (CGAAGGCGTCTTGATGGGTTTG) (25 cycles, *T*_a_ = 52 °C) (Supplementary Table 22). Reaction products were separated by agarose gel electrophoresis (Fig. 1d; the uncropped image of the gel is available in the Source Data file). To obtain amplicons derived from extended CRISPR arrays in strain KD403, PCR reactions were performed using primers LDR-F2 (ATGCTTTAAGAACAAATGTATACTTTTAG) and autoSp2_R (AATAGCGAACAACAAGGTCGGTTG) (30 cycles, *T*_a_ = 52 °C) (Supplementary Table 22). Reaction products were separated by agarose gel electrophoresis, and the amplicon derived from the extended array was purified from the gel using a GeneJET Extraction Kit (Thermo Fisher Scientific) and sequenced on a NextSeq 500 system.

Bioinformatic analysis was performed in R using ShortRead and Biostrings packages^27^. Bases with quality <20 were substituted with N and spacer sequences were extracted from the reads containing two or more CRISPR repeats. Spacers of length 33 bp were mapped to the KD403 genome to identify 33-bp protospacer sequences with 0 mismatches. Spacers that aligned to a single position in the chromosome were used to determine protospacer distribution along the genome. Spacers arising from protospacers due to potential slippage or flippage were removed from analysis^31^ (Supplementary Tables 4, 5).

### Prespacer efficiency assay

Prespacer efficiency assay was performed according to the following protocol^17^. Overnight culture of BL21-AI cells containing a plasmid pCas1 + 2 was diluted 30-fold into 9 ml of LB supplemented with 50 μg/ml streptomycin, 13 mM l-(+)-arabinose and 1 mM IPTG and grown at 37 °C for 2 h. Cells were harvested by centrifugation at +4 °C (1 ml of cells per transformation), washed twice with cold water and resuspended in 50 μl of a solution containing 3.125 μM complementary oligonucleotides (Supplementary Table 13). Electroporation was carried out in a 1-mm gap cuvette at a voltage of 1.8 kV. 3 ml of LB supplemented with 50 μg/ml streptomycin was added to the electroporated cells and the cultures were incubated at 37 °C during 2 h. Lysates of cell cultures were prepared and used in PCR reactions containing a primer BLCRdir complementary to the leader sequence (GGTAGATTGTGACTGGCTTAAAAAATC) and a primer BLCRreverse complementary to the preexisting spacer in the array (GTTTGAGCGATGATATTTGTGCTC), respectively (Supplementary Table 22). Amplicons corresponding to extended and nonextended CRISPR arrays were isolated using GeneJET PCR Purification Kit (ThermoFisher Scientifc) and sequenced on a NextSeq 500 platform. Bioinformatic analysis was performed in R using ShortRead and Biostrings packages^27^. Reads containing the bases with Phred quality <14 were removed from analysis and reads containing at least one CRISPR repeat were further analyzed. Newly acquired spacers were extracted from the expanded reads and mapped to the genome, plasmid and transforming oligonucleotide sequence with two mismatches allowed; 33-bp oligo-derived spacers that were cut between AA and G before integration were considered as properly processed. For simplicity, only properly processed oligo-derived spacers inserted into the CRISPR array in direct (GCCCAATTTACTACTCGTTCTGGTGTTTCTCGT) or reverse (ACGAGAAACACCAGAACGAGTAGTAAATTGGGC) orientation were included into analysis.

### Isolation of DNA fragments generated in vivo

Total genomic DNA was isolated from cultures of strains KD403, KD518, KD753, KD263 and KD675 by collecting 1.25 ml of cell suspensions by centrifugation, resuspending cells in 125 μl of PBS, adding 2 ml of lysis buffer (0.6% SDS, 12 μg/ml proteinase K in 1× TE buffer) and incubating at 55 °C for 1 h. Two milliliters of phenol:chloroform:isoamyl alcohol (25:24:1) (pH 8) was added to the lysate, the solution was gently mixed, and the aqueous and organic phases separated by centrifugation at 7000×*g* for 10 min at room temperature. The upper aqueous phase containing total genomic DNA was collected and the residual phenol was removed by the addition of 2 ml of chloroform:isoamyl alcohol (24:1). The solution was gently mixed, centrifuged at 7000×*g* for 10 min at room temperature. The upper DNA-containing fraction was transferred to a fresh tube; 0.2 M NaCl, 15 μg/ml of Glycoblue (Invitrogen) and two volumes of cold 100% ethanol were added, and the solution was incubated at −80 °C overnight. Precipitated DNA was recovered by centrifugation at 21,000×*g* for 30 min at 4 °C. Pellets were washed twice with 80% ethanol, resuspended in 200 μl of 1× TE buffer, and treated with 1 mg/ml RNase A at 37 °C for 30 min to remove the residual RNA. DNA was isolated by phenol:chloroform:isoamyl alcohol extraction and ethanol precipitation as described above.

DNA fragments <700 bp in length were isolated from 9 μg of total genomic DNA using a Select-a-Size DNA Clean & Concentrator kit (Zymo Research) according to manufacturer’s recommendations. To ensure the binding of fragments <50 bp to the column filter, the volume of 100% ethanol added to the fraction prior to on-filter purification was increased from 290 μl to 600 μl. DNA fragments were eluted with 2 × 50 μl of elution buffer, pooled and purified by ethanol precipitation. A total of 100 μl of DNA was mixed with 10 μl of 3 M NaOAc (0.1×V), 1 μl of 10 mg/ml glycogen (0.01×V) and 330 μl of 100% ethanol, vortexed, and incubated overnight at −80 °C. DNA was recovered by centrifugation at 21,000×*g* for 30 min at 4 °C. Pellets were washed three times with 80% cold ethanol, air dried for ~5 min, and resuspended in 5 μl of nuclease-free water.

### High-throughput sequencing of DNA fragments: FragSeq

The DNA oligo i116 that served as a 3′ adapter was adenylated using 5′ DNA Adenylation Kit (NEB), purified by ethanol precipitation as above and diluted to 10 μM with nuclease-free water (Supplementary Table 23).

DNA fragments <700 bp (in 5 μl of water) were heat-denatured at 95 °C for 5 min, cooled to 65 °C, and mixed with 0.5 μM adenylated oligo i116, 1× NEBuffer 1, 5 mM MnCl_2_ and 10 pmol of thermostable 5′ App DNA/RNA ligase (NEB) in 10-μl reaction volume. The mixture was incubated at 65 °C for 1 h, heated at 90 °C for 3 min, and cooled to 4 °C on ice. Ligated products were combined with 1× T4 RNA ligase buffer, 12% PEG 8000, 10 mM DTT, 60 μg/ml BSA and 10 U of T4 RNA ligase 1 (NEB) in a 25-μl reaction volume. The reaction was incubated at 16 °C for 16 h; 25 μl of 2× loading dye was added, and the products were separated by electrophoresis on 10% 7 M urea slab gels (equilibrated and run in 1× TBE buffer). The gel was stained with SYBR Gold nucleic acid gel stain, bands were visualized on a UV transilluminator, and products of ~40 to ~500 nt were excised from the gel and recovered as described in Vvedenskaya et al.^32^. Briefly, the excised gel slice was crushed, 400 μl of 0.3 M NaCl in 1× TE buffer was added, and the mixture incubated at 70 °C for 10 min. The eluate was collected using a Spin-X column. After the first elution step, the elution procedure was repeated, eluates were pooled, and DNA was isolated by ethanol precipitation and resuspended in 15 μl of nuclease-free water.

Next, the 3′ adapter-ligated DNA fragments were adenylated using 5′ DNA Adenylation Kit (NEB) in a 20-μl reaction following the manufacturer’s recommendations. Nuclease-free water was added to 100 μl, DNA fragments were purified by ethanol precipitation and resuspended in 5 μl of nuclease-free water. The two-step ligation procedure described above was repeated using 5 μl of adenylated 3′-ligated DNA fragments, 0.5 μM of barcoded oligos i112, i113, i114 or i115 that served as 5′ adapters (barcodes were used as internal controls; Supplementary Table 23), 10 pmol of thermostable 5′ App DNA/RNA ligase at the first ligation step, and 10 U of T4 RNA ligase 1 at the second ligation step. Reactions were stopped by addition of 25 μl of 2× loading dye, and the products were separated by electrophoresis on 10% 7 M urea slab gels (equilibrated and run in 1× TBE buffer). DNA products of ~70 to ~500 nt in size were excised and eluted from the gel as described above, isolated by ethanol precipitation, and resuspended in 20 μl of nuclease-free water.

To amplify DNA, 2–8 μl of adapter-ligated DNA fragments were added to a mixture containing 1× Phusion HF reaction buffer, 0.2 mM dNTPs, 0.25 μM Illumina RP1 primer, 0.25 μM Illumina index primer and 0.02 U/μl Phusion HF polymerase in a 30-μl reaction (Supplementary Table 24). PCR was performed with an initial denaturation step of 30 s at 98 °C, amplification for 15 cycles (denaturation for 10 s at 98 °C, annealing for 20 s at 62 °C and extension for 15 s at 72 °C), and a final extension for 5 min at 72 °C. Amplicons were isolated by electrophoresis using a non-denaturing 10% slab gel (equilibrated and run in 1× TBE). The gel was stained with SYBR Gold nucleic acid gel stain and species of ~150 to ~300 bp were excised. DNA products were eluted from the gel with 600 μl of 0.3 M NaCl in 1× TE buffer at 37 °C for 3 h, purified by ethanol precipitation, and resuspended in 25 μl of nuclease-free water. Barcoded libraries were sequenced on Illumina NextSeq 500 platform in high output mode.

Bioinformatic analysis was performed in R using ShortRead and Biostrings packages^27^. Bases with quality <20 were substituted with N. After adapter trimming, all reads were compared to each other to reveal clusters of overamplified reads containing the same insert and combination of unique molecular identifiers conjugated to adapters. For each cluster, a consensus sequence was extracted and used together with non-overamplified reads for further alignment to KD403 reference genome with two mismatches allowed. Only reads with a length 16–100 nt uniquely aligned to the genome were further analyzed (Supplementary Fig. 4). Logos were generated using ggseqlogo package^33^.

### Reporting Summary

Further information on research design is available in the Nature Research Reporting Summary linked to this article.

## Supplementary information

Supplementary Information

Peer Review

Reporting Summary

Source Data

## Data Availability

A reporting summary for this Article is available as a Supplementary Information file. Raw sequencing data obtained in this study are available in Sequence Read Archive (BioProject Accession: PRJNA552808). The source data underlying Figs. 1b, d, e, 2a, b, 3b and Supplementary Figs. 1a and 3a are provided as a Source Data file. All data are available from the corresponding author upon reasonable request.

## Code availability

Custom code and information about software used in this study is available at GitHub (https://github.com/AnnaBioLogic/Shiriaeva_et_al_2019).
