# Supplementary material for: Detection of spacer precursors formed in vivo during primed CRISPR adaptation
Source: Nat Commun. 2019 Oct 10;10:4603. doi: 10.1038/s41467-019-12417-w (PMC6787059; doi:10.1038/s41467-019-12417-w)
Supplement: Supplementary file 1 — Supplementary Information [file 41467_2019_12417_MOESM1_ESM.pdf]

Supplementary Information

Detection of Spacer Precursors Formed *In Vivo* During Primed CRISPR Adaptation

Shiriaeva et al.

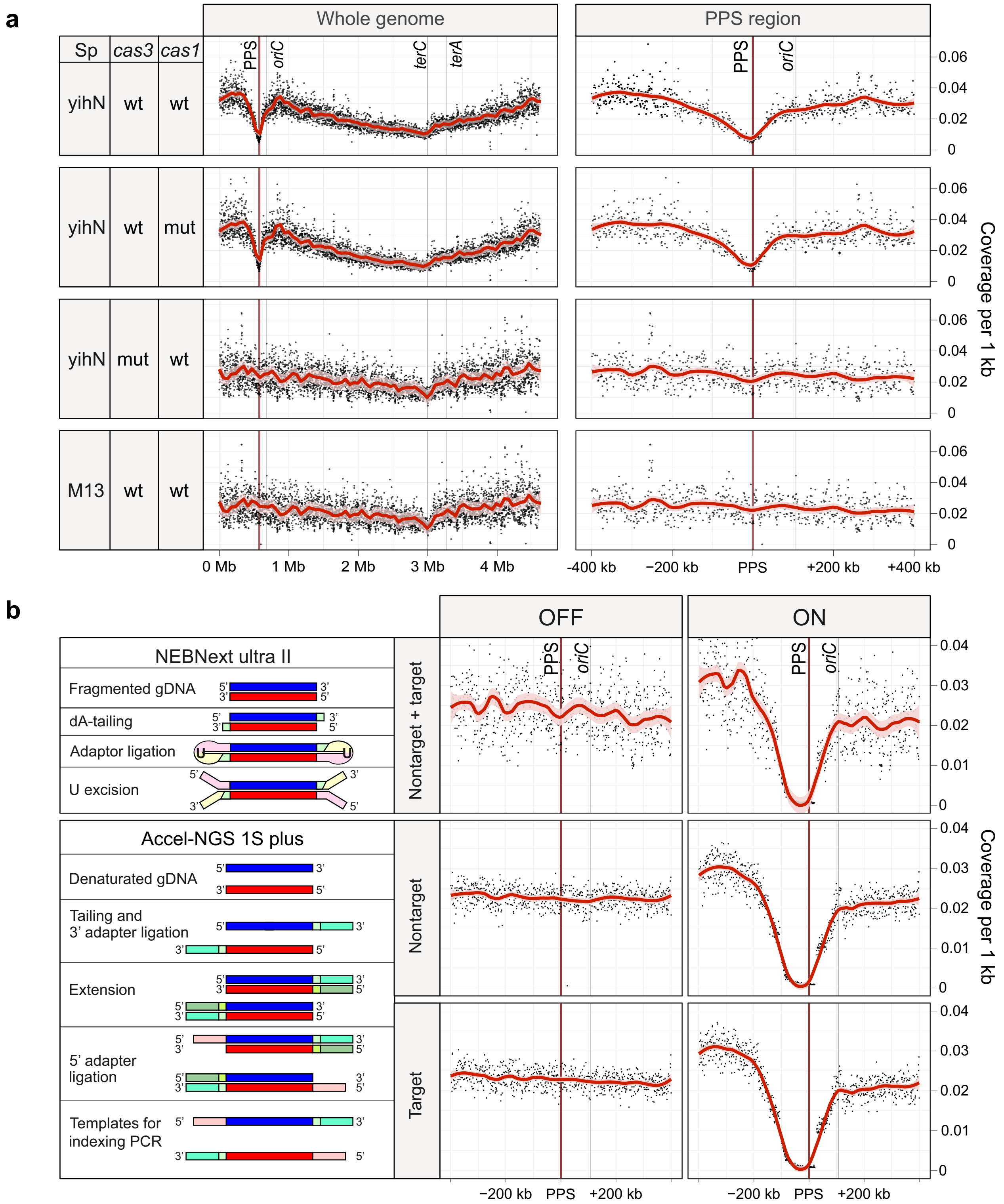

Supplementary Figure 1. CRISPR interference in the type I-E self-targeting system results in loss of chromosomal DNA in the PPS^{yihN} region. a, High-throughput-sequencing analysis of genomic DNA: effects of disruptions in components of interference or adaptation. Graph of sequence coverage per 1 kb for the whole genome (left) or PPS^{yihN} region (right) in the indicated strains. *oriC*, site of replication origin; *terA* and *terC*, sites of replication termination; dot, coverage per 1 kb (mean of 3 biological replicates); red line, Loess smoothing; pink shading, 99% confidence interval. *cas1* mut, gene encoding Cas1^{H208A}, *cas3* mut, gene encoding Cas3^{H74A}. (We note that differences in growth rate likely account for the difference in coverage between *oriC* and the *terC* site in cells undergoing interference vs. cells not undergoing interference; Supplementary Table 3). **b,** High-throughput sequencing analysis of genomic DNA: comparison of library construction methods. Left, steps in library construction using a NEBNext ultra II kit (analysis of double-stranded DNA) or Accel NGS 1S plus kit (analysis of single-stranded DNA). Right, PPS-region coverage plots obtained for wild-type cells.

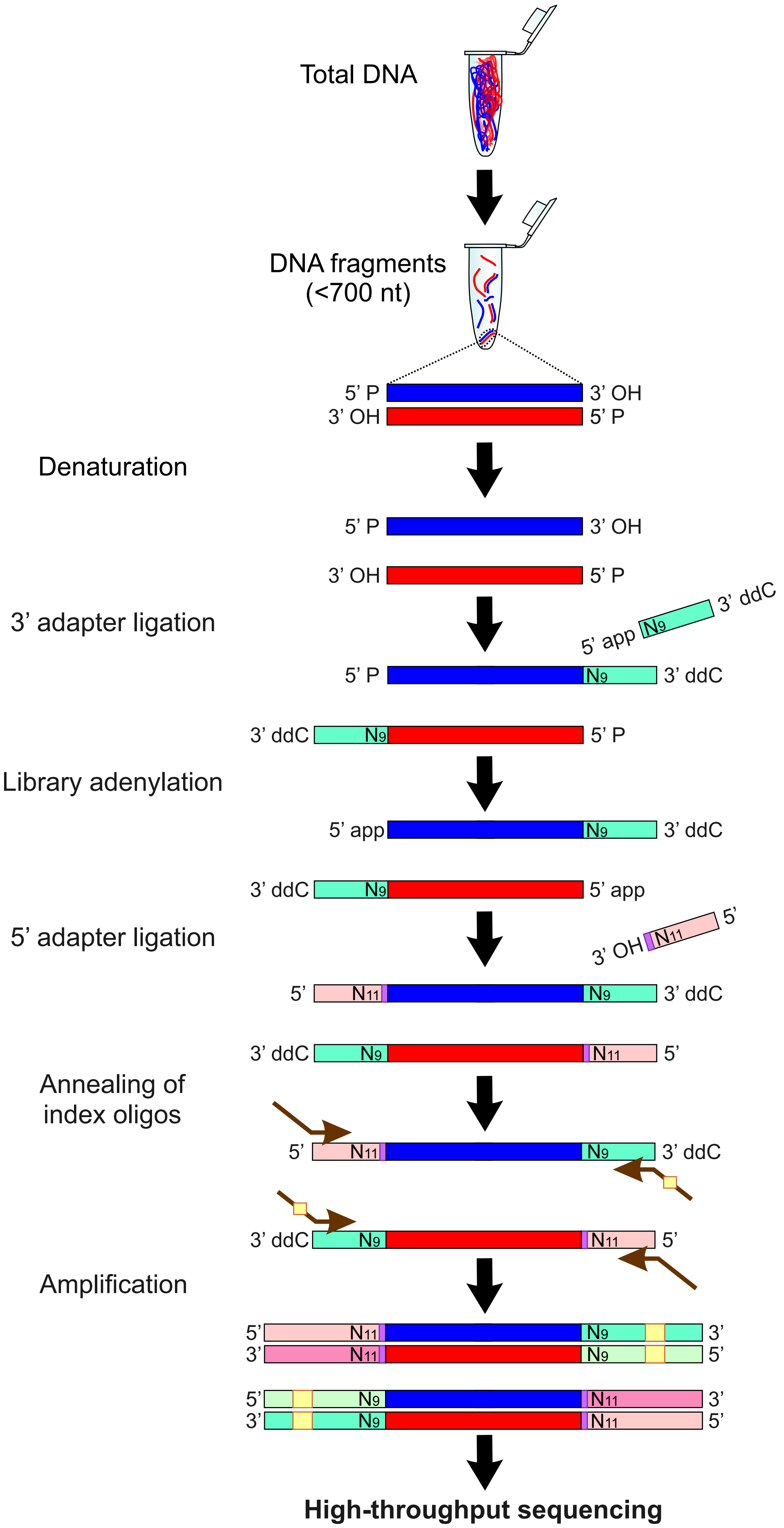

Supplementary Figure 2. Strand-specific, high-throughput sequencing of DNA fragments, FragSeq.
Steps in library construction. 5' app, adenylated 5' end; 3' ddC, blocked 3' end; N9 and N11, unique molecular identifiers on 3' and 5' adapters; purple rectangle, 4-nt barcode on 5' adapter; yellow rectangle, index.

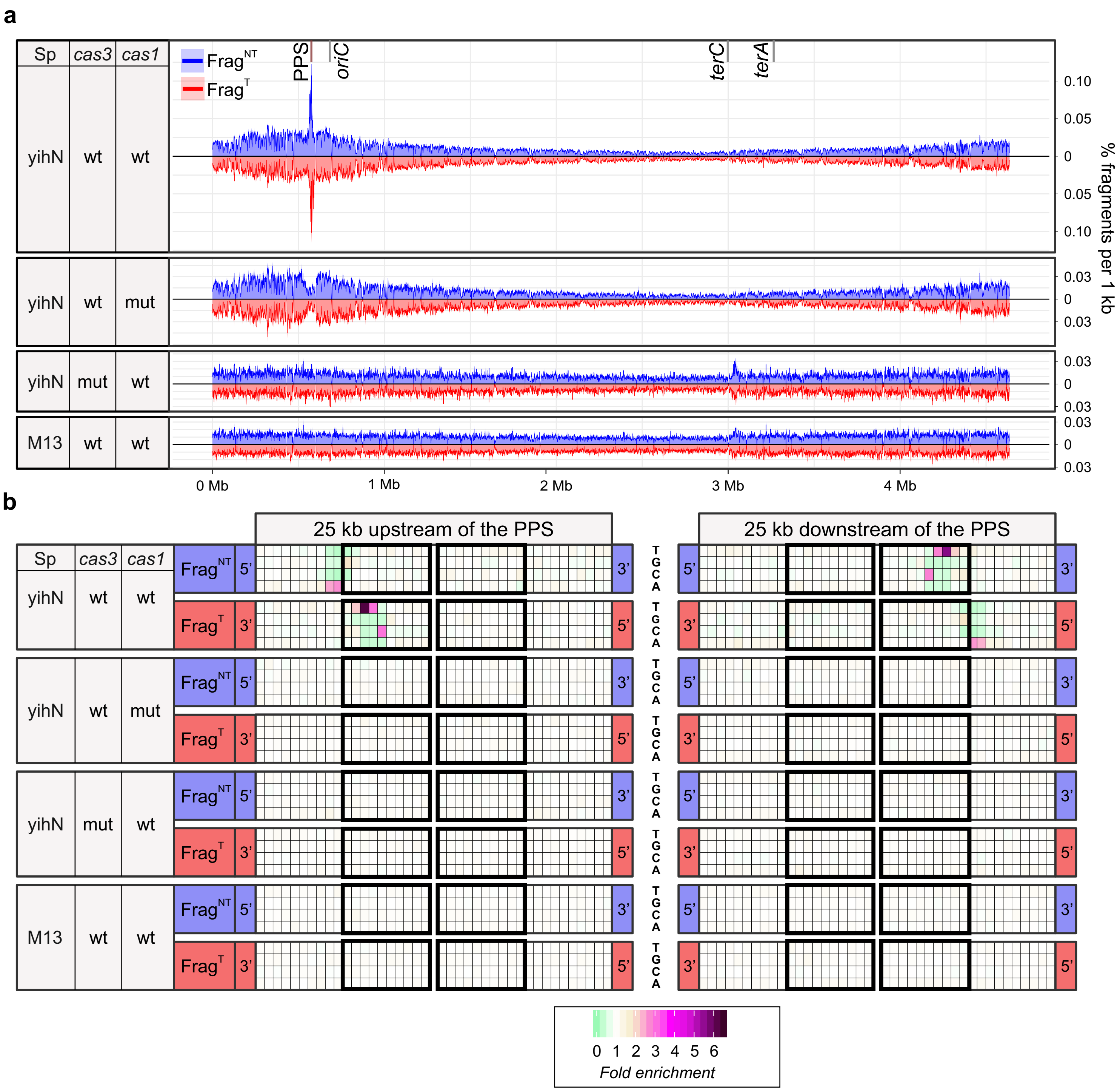

Supplementary Figure 3. FragSeq results for the type I-E self-targeting system: fragment coverage plots and sequence analysis.

a, Genomic coverage plots. Percentage of total DNA fragments per 1 kb for the indicated strains (mean of three biological replicates). Coordinates on the X-axis represent the location on the *E. coli* chromosome. Blue, nontarget-strand-derived fragments (Frag^{NT}); red, target-strand-derived fragments (Frag^{T}). **b,** Sequence features of PPS-region fragments and adjacent chromosomal region. Plot shows heat map of relative abundance of A, T, C, or G for the indicated fragment 5' or 3' ends. Ten positions of sequences that are detected in fragment 5' or 3' ends are shown in black rectangles. Shading represents enrichment (>1) or depletion (<1) of each nucleotide for sequences associated with PPS-region-derived fragments vs. sequences associated with non-PPS-region-derived fragments.

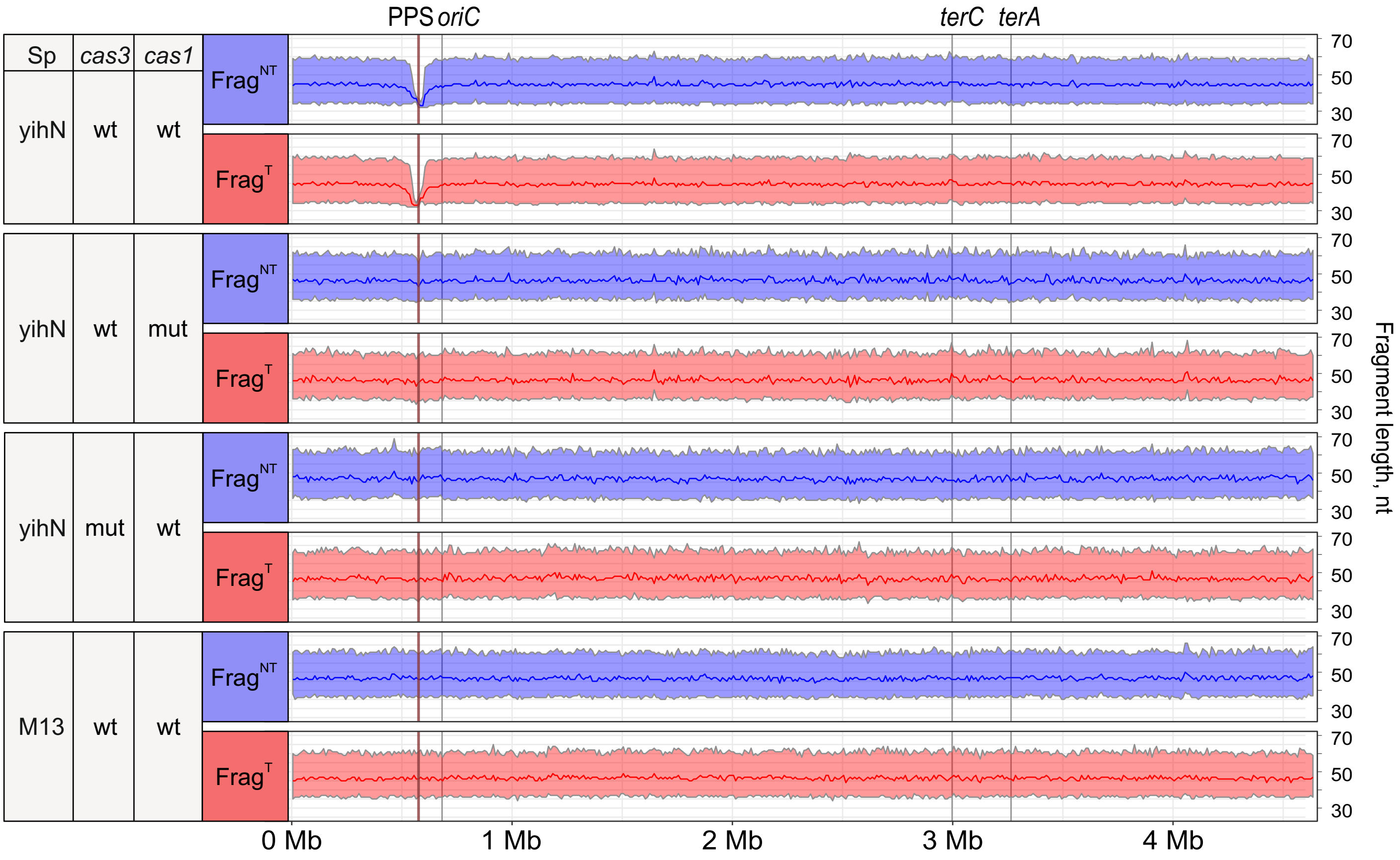

Supplementary Figure 4. FragSeq results for the type I-E self-targeting system: length distributions.
Length distributions of genome-derived fragments in the indicated strains. Coordinates on the X-axis represent the location on the *E. coli* chromosome. Solid lines represent the median fragment length per 10 kb, shaded areas represent fragment lengths between the first and third quartiles.

Supplementary Table 1. Strains used in this study

Name	Description	Source
KD403	K-12 F^+, *lacUV5-cas3 araBp8-cse1*, CRISPR I: repeat-Sp^{yihN}-repeat, CRISPR II deleted. Sp^{yihN} (TCAAACAACCGACCTTGTTGTTCGCTATTGCC) targets chromosomal protospacer PPS (CCAAACAACCGACCTTGTTGTTCGCTATTGCC) within *yihN* gene forming a mismatch between crRNA and PPS at position +1.	This study
KD518	Like KD403, except Cas1 H208A	This study
KD753	Like KD403, except Cas3 H74A	This study
KD263	Like KD403, except CRISPR I: repeat-Sp^{M13}-repeat. Sp^{M13} (CTGTCTTTCGCTGCTGAGGGTGACGATCCCGC) targets g8 gene of M13 phage.	Shmakov *et al.*[1]
BL21-AI	F^- *ompT hsdSB (rB- mB-) gal dcm araB::T7RNAP-tetA*	Invitrogen
KD675	BL21-AI_ΔCRISPR carrying *Pseudomonas aeruginosa* CRISPR array with a single spacer (ACGCAGTTGCTGAGTGTGATCGATGCCATCAG) and a protospacer with a mismatch at position +1 (TCGCAGTTGCTGAGTGTGATCGATGCCATCAG) preceded by a functional GG PAM introduced into *ompL*/*yihN* intergenic region corresponding to the positions 4372171-4372261 of NC_012947	Vorontsova *et al.*[2]

Supplementary Table 2. Statistics for sequencing total genomic DNA purified from self-targeting strain KD403 (with or without induction of *cas* genes expression)

Library preparation	Strain	Number of reads aligned to the genome	Mean coverage, all genome	Mean coverage, PPS-flanking regions*	Mean coverage, PPS	Mean coverage, *terC*	Ratio of coverage: PPS-flanking regions / PPS	Ratio of coverage: PPS-flanking regions / *terC*
NEBNext® Ultra™ II DNA Library Prep Kit (NEB)	KD403 -I	3413501	47.4	50.1	45.6	40.0	1.1	1.3
Accel-NGS® 1S Plus DNA Library Kit (Swift Biosciences)	KD403 -I	4666914	64.8	70.9	70.5	64.2	1.0	1.1
NEBNext® Ultra™ II DNA Library Prep Kit (NEB)	KD403 +I	2117093	48.5	52.0	0.3	38.2	183.8	1.4
Accel-NGS® 1S Plus DNA Library Kit (Swift Biosciences)	KD403 +I	2974368	41.2	47.8	0.2	37.5	251.9	1.3

*Mean coverage over PPS-flanking regions was calculated as a mean of coverage 200 kb upstream and 100 kb downstream of the PPS

Supplementary Table 3. Statistics for sequencing total genomic DNA purified from induced self-targeting strain and control *cas1* mutant (Cas1 H208A), *cas3* mutant (Cas3 H74A) and nontargeting cells

Libraries were prepared only using NEBNext® Ultra™ II DNA Library Prep Kit for Illumina (NEB)

Strain	Replica	Number of reads aligned to the genome	Mean coverage	Mean coverage, PPS-flanking regions*	Mean coverage, PPS	Mean coverage, *terC*	Ratio of coverage: PPS-flanking regions / PPS**	Ratio of coverage: PPS-flanking regions / *terC****
KD263 (nontargeting)	1	7351683	154.6	166.0	147.6	100.8	1.1	1.6
	2	7313371	155.6	166.8	153.1	99.7	1.1	1.7
	3	5953547	123.4	132.9	117.6	79.8	1.1	1.7
KD753 (*cas3* mutant)	1	5826829	122.0	134.5	105.0	77.2	1.3	1.7
	2	1875434	39.0	43.5	33.7	24.7	1.3	1.8
	3	1887489	40.2	44.9	34.8	25.9	1.3	1.7
KD403 (self-targeting)	1	1148014	22.0	32.7	7.0	11.4	4.7	2.9
	2	886656	16.8	23.9	4.5	8.5	5.3	2.8
	3	632137	11.9	16.0	3.2	6.4	5.0	2.5
KD518 (*cas1* mutant)	1	844872	15.9	23.4	5.2	7.3	4.5	3.2
	2	766838	14.6	25.4	7.0	6.6	3.6	3.8
	3	1108980	20.9	33.5	7.1	9.9	4.7	3.4

*Mean coverage over PPS-flanking regions was calculated as a mean of coverage 200 kb upstream and 100 kb downstream of the PPS

**The ratio of genomic coverage between PPS-flanking regions and PPS is greater in self-targeting wild-type or *cas1* mutant strain compared to self-targeting *cas3* mutant or nontargeting cells in which Sp^{yihN} is replaced by a spacer targeting M13 phage (Sp^{M13}) (Cultures capable of interference vs. cultures incapable of interference, p-value = 0.001, Wilcoxon rank sum test).

***The ratio of genomic coverage between region in proximity to the *oriC* and the *terC* sites is greater in self-targeting wild-type or *cas1* mutant strain compared to self-targeting *cas3* mutant or nontargeting cells in which Sp^{yihN} is replaced by a spacer targeting M13 phage (Sp^{M13}) (Cultures capable of interference vs. cultures incapable of interference, p-value = 0.001, Wilcoxon rank sum test).

Supplementary Table 4. Statistics for sequencing spacers acquired during primed adaptation in self-targeting KD403 strain (number of protospacers on each strand upstream or downstream of the PPS)

Replica	Slipped and flipped AAG-protospacers	Number of newly acquired spacers sequenced	% protospacers from total number of protospacers			
			Protospacers in region 100 kb upstream of the PPS		Protospacers in region 100 kb downstream of the PPS	
			Nontarget strand	Target strand	Nontarget strand	Target strand
1	Included	1031147	57.3	1.2	0.8	39.8
	Removed	1005715	57.6	0.8	0.6	40.1
2	Included	1108683	57	1.2	0.9	40.3
	Removed	1079093	57.3	0.8	0.6	40.6
3	Included	1087616	56.4	1.6	1.3	38.9
	Removed	1058208	57	1	0.9	39.3

Supplementary Table 5. Statistics for sequencing spacers acquired during primed adaptation in self-targeting KD403 strain (% protospacers flanked by AAG PAM on each strand upstream or downstream of the PPS)

Replica	Slipped and flipped AAG-protospacers	% protospacers flanked by AAG PAM in the analyzed region			
		Protospacers in region 100 kb upstream of the PPS		Protospacers in region 100 kb downstream of the PPS	
		Nontarget strand	Target strand	Nontarget strand	Target strand
1	Included	95.4	45.1	52.5	95.8
	Removed	97.2	66.8	76.9	97.5
2	Included	95.2	43.3	49.1	95.7
	Removed	97.3	66.4	73.5	97.5
3	Included	96.4	44	56.8	96.7
	Removed	98	74	85.1	98.3

Supplementary Table 6. Statistics for sequencing short DNA fragments generated *in vivo* in type I-E system

Strain	Replica	Amount of reads before removal of overamplified reads	Amount of reads after removal of overamplified reads	Reads uniquely aligned to the genome
KD263 (nontargeting)	1	5694320	991750	730865
	2	1118248	272398	198314
	3	1068441	538644	436247
KD753 (*cas3* mutant)	1	1792903	564591	430276
	2	4061792	797131	619071
	3	765178	200324	130917
KD403 (self-targeting)	1	6329070	1853456	1479620
	2	652932	427896	283221
	3	4193159	1964365	1650564
KD518 (*cas1* mutant)	1	151277	115932	74883
	2	3292762	1155851	897906
	3	247651	211711	130165

Supplementary Table 7. Statistics for sequencing short DNA fragments generated *in vivo* in type I-E system (reads in 50-kb PPS-containing region*)

Strain	Replica	Total number of reads	Reads mapped to the PPS-containing region*	Reads in PPS-containing region with either 5'-CTTNN-3' or 5'-AAG-3' motif**	Reads mapped to the PPS-containing region, % from total	Reads in PPS-containing region with either 5'-CTTNN-3' or 5'-AAG-3' motif**, % from all reads in the PPS-containing region
KD263 (nontargeting)	1	730865	8377	352	1.15	4.20
	2	198314	2401	103	1.21	4.29
	3	436247	5009	219	1.15	4.37
KD753 (*cas3* mutant)	1	430276	5497	342	1.28	6.22
	2	619071	8030	478	1.30	5.95
	3	130917	1619	88	1.24	5.44
KD403 (self-targeting)	1	1479620	69878	44684	4.72	63.95
	2	283221	12105	8031	4.27	66.34
	3	1650564	57947	34529	3.51	59.59
KD518 (*cas1* mutant)	1	74883	841	42	1.12	4.99
	2	897906	10875	551	1.21	5.07
	3	130165	1415	79	1.09	5.58

* PPS-containing region is a region spanning 25 kb upstream and 25 kb downstream of the PPS

**Reads with 5'-CTTNN-3' motif have this motif on their 3' ends; reads with 5'-AAG-3' motif have either 5'-A/AG-3' or 5'-AA/G-3' motif on their 5' ends

Supplementary Table 8. Statistics for sequencing short DNA fragments generated *in vivo* in type I-E system (reads outside of 50-kb PPS-containing region*)

Strain	Replica	Total number of reads	Reads mapped outside of the PPS-containing region*	Reads outside of PPS-containing region* with either 5'-CTTNN-3' or 5'-AAG-3' motif**	Reads outside of PPS-containing region with either 5'-CTTNN-3' or 5'-AAG-3' motif**, % from all reads outside of the PPS-containing region*
KD263 (nontargeting)	1	730865	722488	27395	3.79
	2	198314	195913	7084	3.62
	3	436247	431238	15956	3.70
KD753 (*cas3* mutant)	1	430276	424779	15639	3.68
	2	619071	611041	21928	3.59
	3	130917	129298	4696	3.63
KD403 (self-targeting)	1	1479620	1409742	66930	4.75
	2	283221	271116	12702	4.69
	3	1650564	1592617	73092	4.59
KD518 (*cas1* mutant)	1	74883	74042	3072	4.15
	2	897906	887031	32005	3.61
	3	130165	128750	4803	3.73

* PPS-containing region is a region spanning 25 kb upstream and 25 kb downstream of the PPS
**Reads with 5'-CTTNN-3' motif have this motif on their 3' ends; reads with 5'-AAG-3' motif have either 5'-A/AG-3' or 5'-AA/G-3' motif on their 5' ends

Supplementary Table 9. Statistics for sequencing short DNA fragments generated *in vivo* in type I-E system (reads mapped to the target strand in 25-kb region upstream of the PPS, "25 kb TS up;" or nontarget strand in 25-kb region downstream of the PPS, "25 kb NS dw")

Strain	Replica	Total number of reads	Reads in 25 kb TS up or 25 kb NS dw region	Reads in 25 kb TS up or 25 kb NS dw region, 36-38 nt	Reads in 25 kb TS up or 25 kb NS dw region, 36-38 nt, with 5'-CTTNN-3' motif*	Reads in 25 kb TS up or 25 kb NS dw region, 36-38 nt, with 5'-CTTNN-3' motif*, % from total number of reads	Reads in 25 kb TS up or 25 kb NS dw region, 36-38 nt, with 5'-CTTNN-3' motif*, % from 36-38 nt reads in this region
KD263 (nontargeting)	1	730865	3903	245	3	4.1E-04	1.22
	2	198314	1129	77	0	0.0E+00	0.00
	3	436247	2527	254	0	0.0E+00	0.00
KD753 (*cas3* mutant)	1	430276	2601	233	45	1.0E-02	19.31
	2	619071	3681	328	29	4.7E-03	8.84
	3	130917	760	62	1	7.6E-04	1.61
KD403 (self-targeting)	1	1479620	29055	16134	13953	9.4E-01	86.48
	2	283221	5131	2911	2586	9.1E-01	88.84
	3	1650564	24337	12638	10761	6.5E-01	85.15
KD518 (*cas1* mutant)	1	74883	419	26	1	1.3E-03	3.85
	2	897906	5242	428	5	5.6E-04	1.17
	3	130165	724	54	4	3.1E-03	7.41

*Reads with 5'-CTTNN-3' motif have this motif on their 3' ends

Supplementary Table 10. Statistics for sequencing short DNA fragments generated *in vivo* in type I-E system (reads mapped to the nontarget strand in 25-kb region upstream of the PPS, "25 kb NS up;" or target strand in 25-kb region downstream of the PPS, "25 kb TS dw")

Strain	Replica	Total number of reads	Reads in 25 kb NS up or 25 kb TS dw region	Reads in 25 kb NS up or 25 kb TS dw region, 32-34 nt	Reads in 25 kb NS up or 25 kb TS dw region, 32-34 nt, with 5'-AAG-3' motif*	Reads in 25 kb NS up or 25 kb TS dw region, 32-34 nt, with 5'-AAG-3' motif*, % from total number of reads	Reads in 25 kb NS up or 25 kb TS dw region, 32-34 nt, with 5'-AAG-3' motif*, % from 32-34 nt reads in this region
KD263 (nontargeting)	1	730865	4459	246	10	1.4E-03	4.07
	2	198314	1269	71	2	1.0E-03	2.82
	3	436247	2477	184	3	6.9E-04	1.63
KD753 (*cas3* mutant)	1	430276	2867	217	56	1.3E-02	25.81
	2	619071	4271	320	87	1.4E-02	27.19
	3	130917	839	58	11	8.4E-03	18.97
KD403 (self-targeting)	1	1479620	40665	23868	21573	1.5E+00	90.38
	2	283221	6937	4166	3846	1.4E+00	92.32
	3	1650564	33479	18777	16994	1.0E+00	90.50
KD518 (*cas1* mutant)	1	74883	420	54	4	5.3E-03	7.41
	2	897906	5523	448	96	1.1E-02	21.43
	3	130165	676	53	12	9.2E-03	22.64

*Reads with 5'-AAG-3' motif have either 5'-A/AG-3' or 5'-AA/G-3' motif on their 5' ends

Supplementary Table 11. Correlation between 32- to 34-nt 5’-AAG-3’-associated fragments and 36- to 38-nt 5’-CTT-3’ associated fragments in self-targeting KD403 strain

	**Fragments with either 5'-A/AG-3' or 5'-AA/G-3' motif on their 5' ends**
**Fragments with 5'-CTTNN-3' motif on their 3' ends**	r=0.48 (95% confidence interval: 0.45-0.51); t = 27.667, df = 2538, p-value < 2.2e-16

Supplementary Table 12. Correlation between number of spacers and corresponding prespacers (DNA fragments conjugated to respective PAM) in self-targeting KD403 strain

	**32- to 34-nt fragments with either 5'-A/AG-3' or 5'-AA/G-3' motif on their 5' ends**	**36- to 38-nt fragments with 5'-CTTNN-3' motif on their 3' ends**
**Spacers**	r=0.57 (95% confidence interval: 0.54-0.59); t = 36.772, df = 2826, p-value < 2.2e-16	r=0.5 (95% confidence interval: 0.48-0.53); t = 30.976, df = 2826, p-value < 2.2e-16

Supplementary Table 13. Oligonucleotides used for prespacer efficiency assay

#	Transforming oligo names	Transforming oligo sequences
1.	G_33<br>C_33	5’GCCCAATTTACTACTCGTTCTGGTGTTTCTCGT 3’<br>3’CGGGTTAAATGATGAGCAAGACCACAAAGAGCA 5’
2.	AAG_35<br>TTC_35	5’ AAGCCCAATTTACTACTCGTTCTGGTGTTTCTCGT 3’<br>3’ TTCGGGTTAAATGATGAGCAAGACCACAAAGAGCA 5’
3.	G_33<br>AGTTC_37	5’ GCCCAATTTACTACTCGTTCTGGTGTTTCTCGT 3’<br>3’ AGTTCGGGTTAAATGATGAGCAAGACCACAAAGAGCA 5’
4.	AG_34<br>AGTTC_37	5’ AGCCCAATTTACTACTCGTTCTGGTGTTTCTCGT 3’<br>3’ AGTTCGGGTTAAATGATGAGCAAGACCACAAAGAGCA 5’
5.	G_32<br>AGTTC_37	5’ GCCCAATTTACTACTCGTTCTGGTGTTTCTCG 3’<br>3’ AGTTCGGGTTAAATGATGAGCAAGACCACAAAGAGCA 5’
6.	AG_33<br>AGTTC_37	5’ AGCCCAATTTACTACTCGTTCTGGTGTTTCTCG 3’<br>3’ AGTTCGGGTTAAATGATGAGCAAGACCACAAAGAGCA 5’

*****Nucleotides corresponding to the PAM are written in red

Supplementary Table 14. Prespacer efficiency assay (overall level of adaptation and source of new spacers)

Transforming oligo	Replica	Number of CRISPR arrays	Spacers aligned to genome or pCas1+2	Spacers aligned only to oligo	% of CRISPR arrays elongated due to incorporation of oligo-derived spacer	% of CRISPR arrays elongated due to incorporation of a spacer from the genome or pCas1+2
G_33 + C_33	1	519963	38830	3711	0.7	7.5
	2	379132	26417	3122	0.8	7.0
	3	528544	32241	5595	1.1	6.1
AAG_35 + TTC_35	1	1242907	65814	113924	9.2	5.3
	2	845820	53575	92624	11.0	6.3
	3	847249	42347	116434	13.7	5.0
G_33 + AGTTC_37	1	958220	54203	115681	12.1	5.7
	2	860995	49232	110677	12.9	5.7
	3	1062383	52337	147334	13.9	4.9
AG_34 + AGTTC_37	1	813150	38076	88079	10.8	4.7
	2	773041	31635	80185	10.4	4.1
	3	309799	13061	34515	11.1	4.2
G_32 + AGTTC_37	1	530912	30089	18224	3.4	5.7
	2	496235	28562	21304	4.3	5.8
	3	962226	53517	53987	5.6	5.6
AG_33 + AGTTC_37	1	623827	39669	15692	2.5	6.4
	2	911818	47412	20233	2.2	5.2
	3	370459	19469	9912	2.7	5.3

Supplementary Table 15. Prespacer efficiency assay (insertion of properly processed* oligo only)

Transforming oligo	Replica	Number of CRISPR arrays	Properly processed oligo-derived spacers*	% of CRISPR arrays elongated due to incorporation of a properly processed oligo-derived spacer*	Direct orientation**	Reverse orientation**	Direct orientation**, %	Reverse orientation**, %
G_33 + C_33	1	519963	3385	0.7	1973	1412	58.3	41.7
	2	379132	2878	0.8	1690	1188	58.7	41.3
	3	528544	5140	1.0	2984	2156	58.1	41.9
AAG_35 + TTC_35	1	1242907	103392	8.3	102624	768	99.3	0.7
	2	845820	85060	10.1	84522	538	99.4	0.6
	3	847249	105799	12.5	105173	626	99.4	0.6
G_33 + AGTTC_37	1	958220	86771	9.1	86339	432	99.5	0.5
	2	860995	78527	9.1	78292	235	99.7	0.3
	3	1062383	100267	9.4	99950	317	99.7	0.3
AG_34 + AGTTC_37	1	813150	71646	8.8	71345	301	99.6	0.4
	2	773041	64577	8.4	64380	197	99.7	0.3
	3	309799	26567	8.6	26513	54	99.8	0.2
G_32 + AGTTC_37	1	530912	1875	0.4	1695	180	90.4	9.6
	2	496235	633	0.1	546	87	86.3	13.7
	3	962226	1328	0.1	1147	181	86.4	13.6
AG_33 + AGTTC_37	1	623827	1918	0.3	1639	279	85.5	14.5
	2	911818	1101	0.1	941	160	85.5	14.5
	3	370459	376	0.1	318	58	84.6	15.4

***** We define properly processed oligos as the oligos that were processed between an A and a G in the PAM sequence (T and C in PAM-complementary sequence) and integrated as a 33 bp spacer

Properly processed oligos can be integrated in direct (spacer starts with G; **GCCCAATTTACTACTCGTTCTGGTGTTTCTCGT) or reverse (spacer ends with C; ACGAGAAACACCAGAACGAGTAGTAAATTGGG**C**) orientation

Supplementary Table 16. Prespacer efficiency assay (length of oligo-derived spacers)

Transforming oligo	Replica	% oligo-derived spacers of 33 bp length
G_33 + C_33	1	91.2
	2	92.3
	3	91.9
AAG_35 + TTC_35	1	91.5
	2	93.1
	3	92.2
G_33 + AGTTC_37	1	91.2
	2	91.8
	3	90.7
AG_34 + AGTTC_37	1	90.8
	2	91.4
	3	90.7
G_32 + AGTTC_37	1	87.8
	2	87.6
	3	87
AG_33 + AGTTC_37	1	88.5
	2	88.6
	3	86.8

Supplementary Table 17. Statistics for sequencing short DNA fragments generated *in vivo* in type I-F system

Strain	Replica	Amount of reads before removal of overamplified reads	Amount of reads after removal of overamplified reads	Reads uniquely aligned to the genome
KD675 (*E.coli* strain with type I-F self-targeting system)	1	12437786	9739694	7034061
	2	12256224	9792344	7128753

Supplementary Table 18. Statistics for sequencing short DNA fragments generated *in vivo* in type I-F system (reads mapped to the target strand in 5-kb region upstream of the PPS, "5 kb TS up;" or nontarget strand in 5-kb region downstream of the PPS, "5 kb NS dw")

Strain	Replica	Total number of reads	Reads in 5 kb TS up or 5 kb NS dw region	Reads in 5 kb TS up or 5 kb NS dw region, 31-32 nt	Reads in 5 kb TS up or 5 kb NS dw region, 31-32 nt, with 5'-CCA-3' motif*	Reads in 5 kb TS up or 5 kb NS dw region, 31-32 nt, with 5'-CCA-3' motif*, % from 31-32 nt reads in this region
KD675 (*E.coli* strain with type I-F self-targeting system)	1	7034061	5740	696	454	**65.23**
	2	7128753	2665	280	145	**51.79**

*Reads with 5'-CCA-3' motif have 5'CC/A-3' motif on their 5' ends

Supplementary Table 19. Statistics for sequencing short DNA fragments generated *in vivo* in type I-F system (reads mapped to the nontarget strand in 5-kb region upstream of the PPS, "5 kb NS up;" or target strand in 5-kb region downstream of the PPS, "5 kb TS dw")

**Strain**	**Replica**	**Total number of reads**	**Reads in 5 kb NS up or 5 kb TS dw region**	**Reads in 5 kb NS up or 5 kb TS dw region, 37-38 nt**	**Reads in 5 kb NS up or 5 kb TS dw region, 37-38 nt, with 5'-TGGNNN-3' motif***	**Reads in 5 kb NS up or 5 kb TS dw region, 37-38 nt, with 5'-TGGNNN-3' motif*, % from 37-38 nt reads in this region**
KD675 (*E.coli* strain with type I-F self-targeting system)	1	7034061	5325	751	260	**34.62**
	2	7128753	2520	383	123	**32.11**

*Reads with 5'-TGGNNN-3' motif have this motif on their 3' ends

Supplementary Table 20. Statistics for sequencing short DNA fragments generated *in vivo* in type I-F system (reads mapped outside of the 10-kb PPS-containing region; 31-32 nt reads)

Strain	Replica	Total number of reads	Reads outside of the PPS-containing region	Reads outside of the PPS-containing region, 31-32 nt	Reads outside of the PPS-containing region, 31-32 nt, with 5'-CCA-3' motif*	Reads outside of the PPS-containing region, 31-32 nt, with 5'-CCA-3' motif*, % from 31-32 nt reads in this region
KD675 (*E.coli* strain with type I-F self-targeting system)	1	7034061	7022770	288270	16846	**5.84**
	2	7128753	7123339	275324	18138	**6.59**

*Reads with 5'-CCA-3' motif have 5'CC/A-3' motif on their 5' ends

Supplementary Table 21. Statistics for sequencing short DNA fragments generated *in vivo* in type I-F system (reads mapped outside of the 10-kb PPS-containing region; 37-38 nt reads)

Strain	Replica	Total number of reads	Reads outside of the PPS-containing region	Reads outside of the PPS-containing region, 37-38 nt	Reads outside of the PPS-containing region, 37-38 nt, with 5'-TGGNNN-3' motif*	Reads outside of the PPS-containing region, 37-38 nt, with 5'-TGGNNN-3' motif*, % from 37-38 nt reads in this region
KD675 (*E.coli* strain with type I-F self-targeting system)	1	7034061	7022770	387537	9720	**2.51**
	2	7128753	7123339	422796	9866	**2.33**

*Reads with 5'-TGGNNN-3' motif have this motif on their 3' ends

Supplementary Table 22. List of primers used for amplification of CRISPR array.

Name	Sequence (5' to 3')	Purpose
LDR-F2	ATGCTTTAAGAACAAATGTATACTTTTAG	Monitoring primed adaptation in KD263 and KD403
Ec_minR	CGAAGGCGTCTTGATGGGTTTG	
LDR-F2	ATGCTTTAAGAACAAATGTATACTTTTAG	High-throughput sequencing of spacers acquired during primed adaptation in KD403
autoSp2_R	AATAGCGAACAACAAGGTCGGTTG	
BLCRdir	GGTAGATTGTGACTGGCTTAAAAAATC	High-throughput sequencing of spacers acquired during prespacer efficiency assay in BL21-AI
BLCRreverse	GTTTGAGCGATGATATTTGTGCTC	

Supplementary Table 23. List of adapters used for FragSeq

Name	Sequence (5' to 3')	Description
i112	GTTCAGAGTTCTACAGTCCGACGATCCTGANNNNNNNNNNN	5′ adapter with CTGA barcode and 11N extension used in KD263 short DNA fragments library preparation (barcode is underlined)
i113	GTTCAGAGTTCTACAGTCCGACGATCGACTNNNNNNNNNNN	5′ adapter with GACT barcode and 11N extension used in KD753 short DNA fragments library preparation (barcode is underlined)
i114	GTTCAGAGTTCTACAGTCCGACGATCAGTCNNNNNNNNNNN	5′ adapter with AGTC barcode and 11N extension used in KD403 short DNA fragments library preparation (barcode is underlined)
i115	GTTCAGAGTTCTACAGTCCGACGATCTCAGNNNNNNNNNNN	5′ adapter with TCAG barcode and 11N extension used in KD518 and KD675 short DNA fragments library preparation (barcode sequence is underlined)
i116	Phos/NNNNNNNNNTGGAATTCTCGGGTGCCAAGG/ddC/	3′ adapter with 9N random sequence used in short DNA fragments library preparation

Supplementary Table 24. List of Illumina primers used for amplification of FragSeq libraries.

Name	Sequence (5' to 3')	Sample
RP1	AATGATACGGCGACCACCGAGATCTACACGTTCAGAGTTCTACAGTCCGA	All samples
RPI3	CAAGCAGAAGACGGCATACGAGAT**GCCTAA**GTGACTGGAGTTCCTTGGCACCCGA GAATTCCA	KD263, replicate 1
RPI4	CAAGCAGAAGACGGCATACGAGAT**TGGTCA**GTGACTGGAGTTCCTTGGCACCCGA GAATTCCA	KD263, replicate 2
RPI5	CAAGCAGAAGACGGCATACGAGAT**CACTGT**GTGACTGGAGTTCCTTGGCACCCGA GAATTCCA	KD263, replicate 3
RPI6	CAAGCAGAAGACGGCATACGAGAT**ATTGGC**GTGACTGGAGTTCCTTGGCACCCGA GAATTCCA	KD753, replicate 1
RPI7	CAAGCAGAAGACGGCATACGAGAT**GATCTG**GTGACTGGAGTTCCTTGGCACCCGA GAATTCCA	KD753, replicate 2
RPI8	CAAGCAGAAGACGGCATACGAGAT**TCAAGT**GTGACTGGAGTTCCTTGGCACCCGA GAATTCCA	KD753, replicate 3
RPI9	CAAGCAGAAGACGGCATACGAGAT**CTGATC**GTGACTGGAGTTCCTTGGCACCCGA GAATTCCA	KD403, replicate 1
RPI10	CAAGCAGAAGACGGCATACGAGAT**AAGCTA**GTGACTGGAGTTCCTTGGCACCCGA GAATTCCA	KD403, replicate 2
RPI11	CAAGCAGAAGACGGCATACGAGAT**GTAGCC**GTGACTGGAGTTCCTTGGCACCCG AGAATTCCA	KD403, replicate 3
RPI12	CAAGCAGAAGACGGCATACGAGAT**TACAAG**GTGACTGGAGTTCCTTGGCACCCGA GAATTCCA	KD518, replicate 1
RPI13	CAAGCAGAAGACGGCATACGAGAT**TTGACT**GTGACTGGAGTTCCTTGGCACCCGA GAATTCCA	KD518, replicate 2
RPI14	CAAGCAGAAGACGGCATACGAGAT**GGAACT**GTGACTGGAGTTCCTTGGCACCCG AGAATTCCA	KD518, replicate 3
RPI20	CAAGCAGAAGACGGCATACGAGAT**GGCCAC**GTGACTGGAGTTCCTTGGCACCCG AGAATTCCA	KD675 replicate 1
RPI21	CAAGCAGAAGACGGCATACGAGAT**CGAAAC**GTGACTGGAGTTCCTTGGCACCCG AGAATTCCA	KD675 replicate 2

PCR was performed with TruSeq Small RNA RP1 primer and one of RPI index primers (index sequence is shown in bold).

Supplementary References

1. Shmakov, S. et al. Pervasive generation of oppositely oriented spacers during CRISPR adaptation. Nucleic Acids Res 42, 5907-5916, (2014).
2. Vorontsova, D. et al. Foreign DNA acquisition by the I-F CRISPR-Cas system requires all components of the interference machinery. Nucleic Acids Res 43, 10848-10860, (2015).
