# Supplementary material for: Detection of spacer precursors formed in vivo during primed CRISPR adaptation
Source: Nat Commun. 2019 Oct 10;10:4603. doi: 10.1038/s41467-019-12417-w (PMC6787059; doi:10.1038/s41467-019-12417-w)
Supplement: Supplementary file 2 — Peer Review [file 41467_2019_12417_MOESM2_ESM.pdf]

Editorial Note: This manuscript has been previously reviewed at another journal that is not operating a transparent peer review scheme. This document only contains reviewer comments and rebuttal letters for versions considered at *Nature Communications.*

REVIEWERS' COMMENTS:

Reviewer #1 (Remarks to the Author):

I previously reviewed this paper and I feel that the authors have effectively addressed all of my queries.

Reviewer #2 (Remarks to the Author):

I appreciate the author's efforts to carefully address the reviewer's concerns. My concerns have been fully addressed. I recommend the publication of this manuscript as is.

Reviewer #3 (Remarks to the Author):

The authors were very receptive to the previous comments and suggestions. This is a well performed study that will of be of broad interest. All conclusions are well supported by the available data. I highly recommend publication in Nature Communications.
